# P-2104. A Quality Improvement Project that Implements Multi-disciplinary Residency Training on Point-of-Care (POCUS) Assistance for Lumbar Punctures (LP) in Infants < 6 Months

**DOI:** 10.1093/ofid/ofae631.2260

**Published:** 2025-01-29

**Authors:** Nathaniel Locke, Daniel Glenn, Connor Kelly, Aicha Hull, Rebecca Sainato

**Affiliations:** Madigan Army Medical Center, Lacey, Washington; Madigan Army Medical Center Pediatrics, University Place, Washington; Madigan Army Medical Center, Lacey, Washington; Madigan Army Medical Center, Lacey, Washington; Madigan Army Medical Center, Lacey, Washington

## Abstract

**Background:**

LPs are critical in the work-up febrile infants to evaluate for meningitis. National data suggests many clinicians struggle to perform this procedure successfully which leads to additional procedures, delayed treatment, and unnecessary hospitalization/antimicrobials. There is evidence that suggests POCUS assistance leads to greater LP success as it is uniquely suited to visualize the infant spine in great detail, which can provide critical information (Figure 1). By increasing POCUS application and awareness, we hope to obtain cerebral spinal fluid (CSF) from > 90% of infants < 6 months within 3 attempts, and before antibiotics in > 75% of cases.

**Figure:**
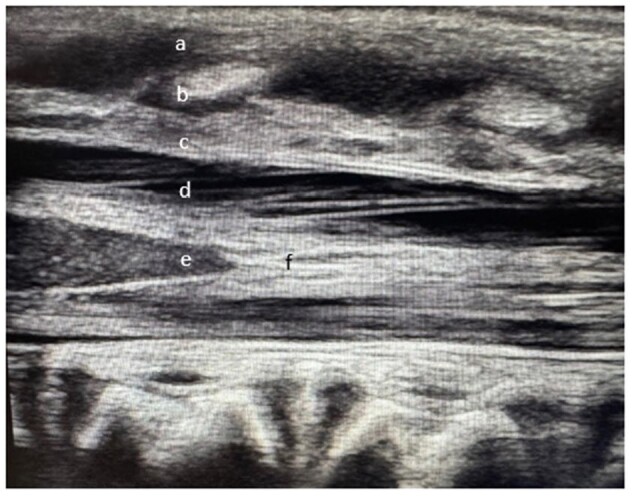

**Methods:**

Utilizing our institution’s electronic medical record (EMR), we reviewed all infants < 6 months admitted to the pediatric ward who had an LP from January 2021 to June 2023 to establish our baseline. Hands-on and didactic training on POCUS-assistance for LP in neonates were provided to multiple residency faculty, and training was integrated within each programs’ curriculum. We created a post-procedural survey and perform quarterly EMR reviews of our progress.

**Figure:**
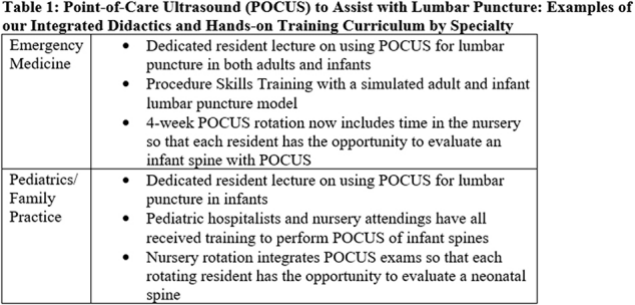

**Results:**

Baseline: CSF was obtained in 33/35 infants (94%). The average number of attempts was 3, interquartile range (IQR) 1-3, and max 10. 14/35 infants (40%) received antibiotics before a sample was obtained. The average time from antibiotics to obtaining an LP was 8.8 hours, IQR 1.4-6.2 hours. 16 infants (46%) had either a traumatic LP or no CSF obtained, including 7 (20%) where LP occurred following antibiotics.

Post-implementation: All interns in family practice, pediatrics, and emergency medicine now receive training on POCUS-assistance for obtaining LPs in infants (Table 1). 7 surveys have been completed and 3 infants were admitted to our ward with concern for meningitis. CSF was obtained in all 3 prior to antibiotics and within 3 attempts (Table 2).

**Figure:**
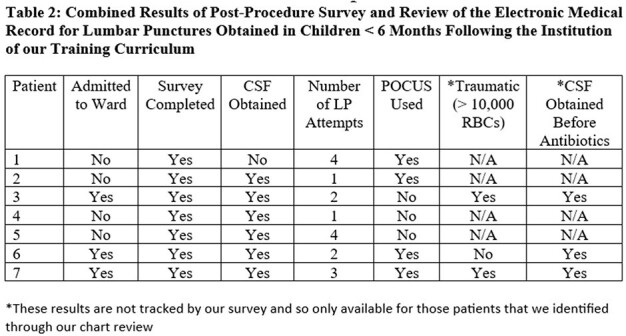

**Conclusion:**

Our EMR review is congruent with national data, suggesting a high rate of LP failure in infants. A new multi-disciplinary curriculum has been established at our hospital and POCUS assistance is now routinely being used for obtaining LPs in those < 6 months. While we have limited post-implementation data, our results show promise towards increasing success, reducing attempts, and decreasing time to antibiotics.

**Disclosures:**

All Authors: No reported disclosures

